# Analysis of insertions and extensions in the functional evolution of the ribonucleotide reductase family

**DOI:** 10.1002/pro.4483

**Published:** 2022-12

**Authors:** Audrey A. Burnim, Da Xu, Matthew A. Spence, Colin J. Jackson, Nozomi Ando

**Affiliations:** ^1^ Department of Chemistry and Chemical Biology Cornell University Ithaca New York USA; ^2^ Research School of Chemistry Australian National University Canberra Australian Capital Territory Australia; ^3^ Australian Research Council Centre of Excellence for Innovations in Peptide and Protein Science Australian National University Canberra Australian Capital Territory Australia; ^4^ Australian Research Council Centre of Excellence in Synthetic Biology Australian National University Canberra Australian Capital Territory Australia

**Keywords:** allostery, evolution, evo‐velocity, structure prediction

## Abstract

Ribonucleotide reductases (RNRs) are used by all free‐living organisms and many viruses to catalyze an essential step in the *de novo* biosynthesis of DNA precursors. RNRs are remarkably diverse by primary sequence and cofactor requirement, while sharing a conserved fold and radical‐based mechanism for nucleotide reduction. In this work, we expand on our recent phylogenetic inference of the entire RNR family and describe the evolutionarily relatedness of insertions and extensions around the structurally homologous catalytic barrel. Using evo‐velocity and sequence similarity network (SSN) analyses, we show that the N‐terminal regulatory motif known as the ATP‐cone domain was likely inherited from an ancestral RNR. By combining SSN analysis with AlphaFold2 predictions, we also show that the C‐terminal extensions of class II RNRs can contain folded domains that share homology with an Fe‐S cluster assembly protein. Finally, using sequence analysis and AlphaFold2, we show that the sequence motif of a catalytically essential insertion known as the finger loop is tightly coupled to the catalytic mechanism. Based on these results, we propose an evolutionary model for the diversification of the RNR family.

## 1 INTRODUCTION

Ribonucleotide reductases (RNRs) are an ancient enzyme family that catalyzes the conversion of ribonucleotides to 2′‐deoxyribonucleotides. They are present in all free‐living organisms and thus have adapted to diverse living environments.^1^ One environmental parameter, the presence of oxygen, has shown to be a determining factor in the diversification of RNRs, as it dictates the mechanism through which the radical involved in catalysis can be generated. Biochemically, three distinct classes of RNRs have been identified. Class I RNRs require oxygen to generate the initial radical in a separate ferritin subunit (known as β). This initial radical is passed onto the catalytic subunit (known as α) during turnover through proton‐coupled electron transfer via the formation of a transient protein complex. In contrast, class III RNRs must generate their radical in an oxygen‐free environment, as both the glycyl radical intermediate and their associated activase (which utilizes radical *S*‐adenosylmethionine [AdoMet] chemistry) are oxygen‐sensitive. Class II RNRs, on the other hand, are neither sensitive nor dependent on oxygen, as they generate a 5′‐deoxyadenosyl radical (5′‐dAdo•) via the homolysis of the cobalt‐carbon bond in their adenosylcobalamin (AdoCbl) cofactor.

**FIGURE 1:**
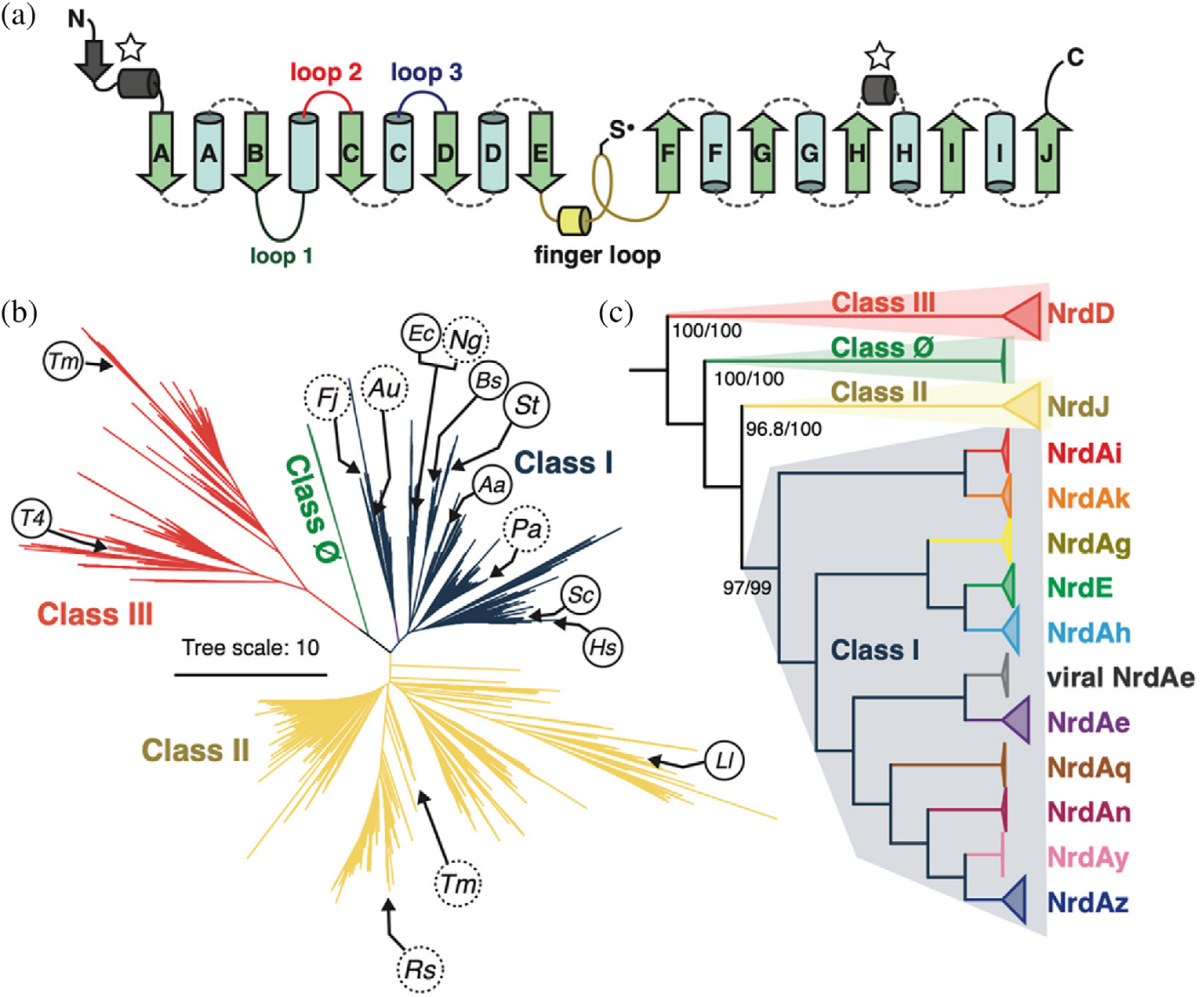
Phylogeny of the ribonucleotide reductase (RNR) family. (a) RNRs share a common barrel topology composed of two halves, each consisting of a 5‐stranded β‐sheet (βA‐βE and βF‐βJ), that are connected by the so‐called “finger loop” (yellow), which contains a conserved cysteine that has been shown to be the site of the catalytically essential thiyl radical in all biochemically characterized RNRs. RNRs are diversified by N‐ and C‐terminal extensions and insertions between the secondary structure elements in the catalytic barrel. Loops 1–3 (dark green, red, blue) are involved in allosteric regulation, and the gray secondary structure elements are involved in substrate binding (starred). (b) Previously published unrooted phylogeny of 6,779 RNR α sequences.^3^ Host organisms of structurally characterized RNRs are shown in circles. In clockwise order, class I: *Fj, Flavobacterium johnsoniae; Au, Actinobacillus ureae; Ec, Escherichia coli*; *Ng, Neisseria gonorrhea; Bs, Bacillus subtilis; St: Salmonella typhimurium; Aa, Aquifex aeolicus; Pa, Pseudomonas aeruginosa*; *Sc, Saccharomyces cerevisiae; Hs, Homo sapiens*; class II: *Ll, Lactobacillus leichmannii; Tm, Thermotoga maritima; Rs, Rhodobacter sphaeroides*; and class III: *T4*, Bacteriophage T4; *Tm, Thermotoga maritima*. The exact sequences for organisms in dashed circles are not in the tree, however their approximate locations are represented by sequences with ≥80% sequence identity. (c) The same phylogeny shown in panel (b), rooted at the midpoint and collapsed according to major clades. Gene‐based nomenclature^4^ is shown.

Despite large differences in the generation and identity of the initial radical, the overall RNR mechanism is highly conserved.^2^ The initial radical is first transferred to a conserved cysteine in the active site of the catalytic subunit (α) to produce a thiyl radical. Nucleotide reduction is then carried out by the thiyl radical along with other active‐site residues serving as proton acceptor/donor or reducing equivalents. The oxidized active‐site residues are ultimately returned to their original state by external reductants. This conserved catalytic mechanism is reflected in the high structural homology in the core of all known RNR structures (Figure S1). The core fold is composed of a 10‐stranded α/β barrel, with the thiyl radical on the so‐called “finger loop” that connects the two halves of the barrel (Figure 1a).^5^ This barrel serves as a scaffold for ribonucleotide reduction, with additional diversity and adaptations realized through extensions and insertions about the core structure.

The extensions and insertions in various RNRs are thought to affect the allosteric regulation of substrate specificity and overall activity based on the presence of other nucleotides. In particular, mechanisms to regulate substrate specificity are most integral to the barrel structure. All extant RNRs, with the exception of the monomeric subset of class II RNRs, are thought to dimerize with the αA and αB helices from each monomer forming a four‐helical bundle at the interface (Figure S1a). In class I and II enzymes, these helices dimerize in anti‐parallel fashion such that each end is capped by a functional insertion called loop 1 (Figure 1a and Figure S1b, dark green), which serves as the binding site of specificity‐regulating effectors.^6^, ^7^ This so‐called specificity site is allosterically coupled to the active site via another functional insertion called loop 2 (Figure 1a and Figure S1b, red),^7^ such that the proper balance of nucleotides for DNA synthesis is maintained.^8^, ^9^ Remarkably, in monomeric class II RNRs, the loop 1 insertion is long enough to interact with the αA and αB helices and structurally mimic the dimer interface and specificity site seen in dimeric class II RNRs (Figure S1a).^10^ In class III enzymes, the insertion equivalent to loop 1 is a helix,^11^ which stabilizes an α_2_ dimer with the αA and αB helices of the two monomers in parallel orientation (Figure S1a), and specificity effectors bind in the dimer interface.^12^ Compared with specificity regulation, which appears to be important in most RNRs, overall activity regulation is only associated with RNRs that have an N‐terminal extension with an ATP‐cone motif or recently found truncated versions of this motif that serve as a form of convergent allostery^16^, ^17^ (Figure S1a, orange domains). In all reported cases, these N‐terminal motifs mediate the formation of large oligomeric structures that interfere with the generation of the catalytically essential thiyl radical.

In addition to allosteric regulation, extensions and insertions around the barrel also diversify the catalytic mechanism. The C‐terminus, for instance, is used for enzyme turnover. In class III RNRs, the glycyl radical domain (GRD) is located at the C‐terminus for housing the initial glycyl radical. Class I and certain class II RNRs, on the other hand, are known to have disordered C‐terminal tails with a pair of cysteines that are used for reducing the active site.^18^, ^19^ However, in general, much remains unknown about the functional diversity of RNR C‐termini.

Recently, we reported the first large‐scale phylogenetic inference of the RNR family by using the conserved α/β barrel core to align the highly diverse sequences (Figure 1).^3^ Our inference, consisting of 6,779 α sequences, showed the divergence of the three biochemical classes and additionally identified a small, phylogenetically distinct “class Ø” clade, consisting of the most minimal RNRs known to‐date (Figure S1). In the work presented here, we perform detailed analyses on insertions and extensions around the core barrel to further understand the mechanisms by which the RNRs may have diversified. Using sequence similarity network (SSN) and evo‐velocity analyses, we first describe a novel evolutionary model for the N‐terminal ATP‐cones, with a focus on its loss and gain within class I RNRs. We then describe the discovery of a previously unknown folded domain in long C‐terminal tails of class II RNRs using AlphaFold2 and sequence analysis. Finally, we perform AlphaFold2 and sequence analyses on the finger‐loop region of class III RNRs, which is closely related to the catalytic diversity of this class. These analyses provide further insights into the evolutionary model of RNRs implied by our α phylogeny. Our approach to study the phylogenetic relationships of RNRs via the conserved catalytic barrel and the mechanisms of diversification via the extensions and insertions can be applied to the study of other diverse enzyme families.

## 2 RESULTS

### 2.1 Correspondence of biochemical and phylogenetic classes

The classification of RNRs has been discussed extensively. From a biochemical perspective, they are best classified by the type of radical cofactor used to generate the catalytic thiyl radical (classes I, II, III). In our phylogenetic work,^3^ all 20 inferences (10 replicates each for the sequence evolution models LG + R10 and WAG + R10) reconstructed these major biochemical classes as monophyletic clades (Figure 1b). However, biochemical *subclasses* do not necessarily correspond to phylogenetic clades. Biochemical studies have identified at least five subclasses based on the metal composition of the radical cofactor in the β subunit (Ia, Ib, Ic, Id, Ie),^20^ but there are many more phylogenetic subclades of the class I RNRs (Figure 1c), and phylogenetically distinct RNRs may have independently acquired similar or identical biochemical mechanisms via convergent evolution. Furthermore, as will be discussed in a later section, biochemical classification can vary within a subclade. We will thus follow previously used gene‐based nomenclature (with the prefix *nrd* for ribo **n** ucleotide **r** e **d** uctase)^21^ to describe phylogenetic subclades. By this convention, the α subunits of classes I, II, and III are called NrdA (or NrdE), NrdJ, and NrdD, respectively. Throughout this paper, the inference with the most likely topology at the major branching points is shown (Figure 1b). The process of phylogenetic reconstruction and topology testing has been discussed for this reconstruction previously.^3^

### 2.2 A single origin for the N‐terminal ATP‐cone domains

We first investigated plausible evolutionary mechanisms of RNR diversification via N‐terminal extensions. This region exhibits significant diversity in length due to the number of ATP‐cone motifs, which can vary from 0 to 3.^14^ The canonical ATP‐cone is a ~100‐residue globular domain composed of a four‐helical bundle with a three‐stranded β‐sheet cap (Figure S1a; orange domain in *Escherichia coli* structure). A functional ATP‐cone domain houses an allosteric site that is responsible for regulating overall enzyme activity in response to endogenous ATP and dATP concentrations by mediating protein–protein interactions.^22^ In our phylogeny, 48.7%, 11.3%, and 48.6% of sequences in the class I, II, and III clades, respectively, contain ATP‐cones. As expected, the ATP‐cone motif is entirely absent from the minimal class Ø sequences.^3^ Because class II sequences do not require protein–protein interactions for radical generation, the role of ATP‐cones in this clade is unclear. Only one class II RNR with an ATP‐cone domain has been studied (that of *Thermoplasma acidophilum*), for which activity regulation was not observed.^23^

**FIGURE 2:**
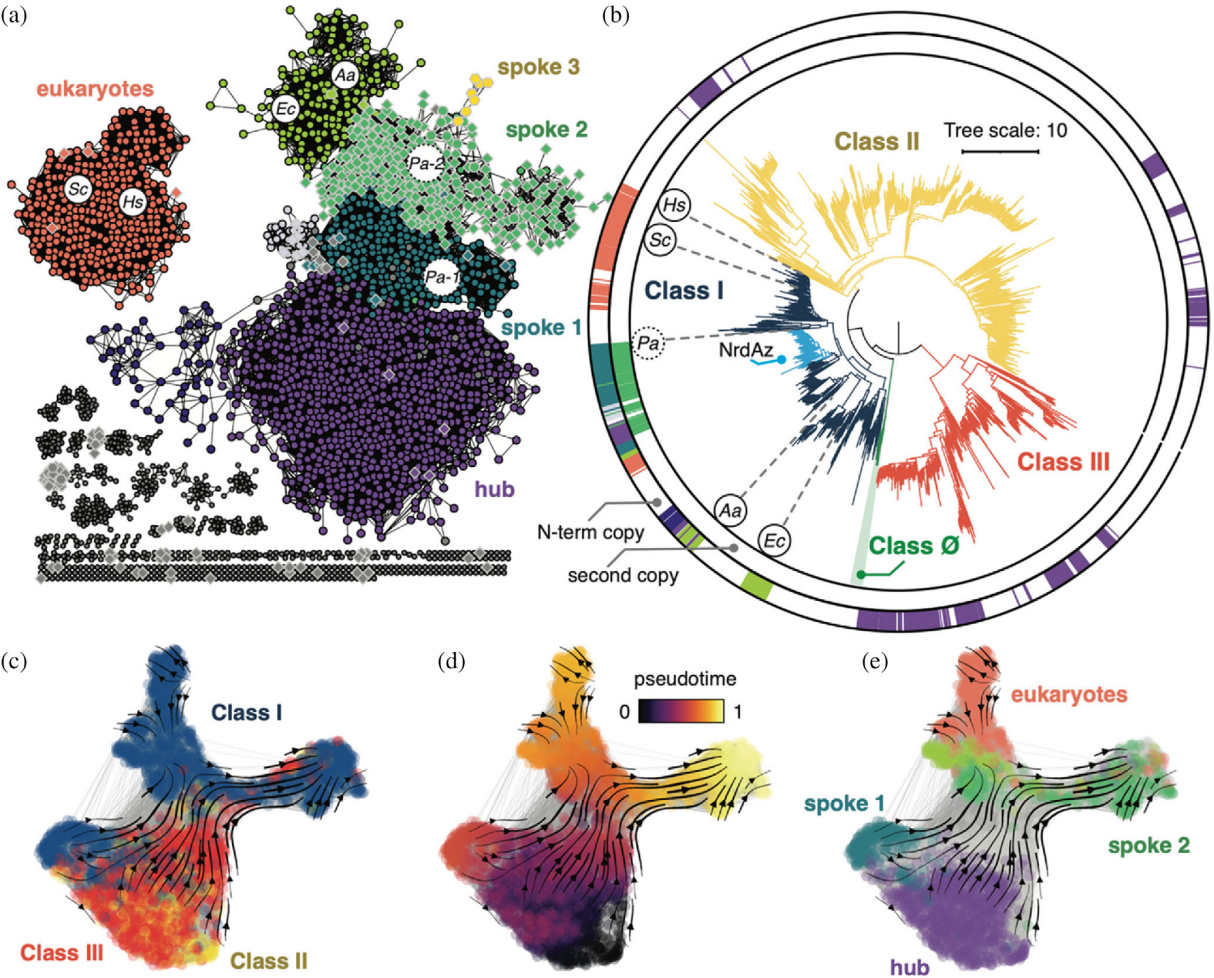
The ATP‐cone domain likely originated from a single origin. (a) A sequence similarity network (SSN) of ATP‐cone domain sequences isolated from 2,653 ɑ sequences in our dataset. Each node is a single ATP‐cone sequence, and each connecting edge indicates an alignment score^24^ greater than 20.5. Circular nodes correspond to the N‐terminal ATP‐cones. Diamond nodes with gray outlines correspond to the second cones in multiple‐ATP‐cone domains, while hexagons with gray outlines correspond to the third. Gray‐filled nodes are not represented on the circumference of the tree in panel B for clarity. (b) Phylogenetic tree from Figure 1, rooted at the midpoint and labeled with color strips corresponding to colors of the major SSN clusters in panel (a). The outer color strips correspond to N‐terminal ATP‐cones (circular nodes in SSN), and the inner color strips correspond to the second copies in multiple‐ATP‐cones (diamond nodes in SSN). Structurally characterized ATP‐cone containing sequences are labeled by host organism: *Ec, Escherichia coli*; *Aa, Aquifex aeolicus*; *Pa, Pseudomonas aeruginosa*; *Sc, Saccharomyces cerevisiae*; *Hs, Homo sapiens*. The exact sequence for *Pa* (dashed circle) is not on the tree, however its approximate location is represented by sequences with 88% sequence identity. (c–e) Evo‐velocity analysis of isolated ATP‐cone sequences. ESM‐1b embedded ribonucleotide reductase (RNR) sequences projected onto a two‐dimensional vector field plot, where the horizontal and vertical axes are UMAP 1 and 2, respectively. Each colored point in the plot corresponds an ATP‐cone sequence. (c) Vector field plot colored by the classification of RNRs from which the ATP‐cone sequence is pruned: class I (blue), class II (yellow), class III (red). (d) Vector field plot colored by pseudotime, a proxy for phylogenetic depth. Indigo (pseudotime = 0) represents ancestral sequences, and yellow (pseudotime = 1) represents sequences that have diverged the most from ancestral sequences. (e) Vector field plot colored by SSN coloring shown in panel (a).

Previous bioinformatic work described the ATP‐cone motif as “evolutionarily mobile” because of its widespread occurrence throughout the RNR family and other proteins.^13^ Despite its connotation, this description is somewhat misleading as there is little evidence that the ATP‐cone has spread via horizontal gene transfer (HGT), and rather little is known about how RNRs may have gained and lost ATP‐cones during evolution. To gain insight into the possible evolutionary history of this motif, we calculated an SSN from isolated ATP‐cone sequences with an alignment score^24^ cutoff of 20.5 (Figure 2a) and mapped major clusters onto the RNR α phylogeny by color (Figure 2b). We observe a “hub‐spoke” topology within our SSN, where clusters (Figure 2a, spokes 1–3) appear to be budding from a large central “hub” of homologous ATP‐cone sequences (Figure 2a, hub) in a sequential manner. Surprisingly, the central hub (Figure 2a, purple nodes) corresponds to ATP‐cone sequences from all three major classes of RNRs (Figure 2b, purple color strips). Moreover, class II and III ATP‐cones remain clustered together in this hub at even more stringent alignment score cutoffs. This unexpected homology suggests a possible shared ancestry and a single origin of the ATP‐cone domain in the last common ancestor (LCA) to the modern RNR.

All three major classes have subclades with multiple ATP‐cones at the N‐terminus, with a particularly high occurrence rate in certain class I subclades (such as the NrdAz clade in Figure 2b). Interestingly, while the central hub mainly consists of single ATP‐cone motifs (Figure 2a, circular nodes in hub), the clusters that bud from this hub (Figure 2a, spokes 1–3) largely correspond to individual copies within multiple‐ATP‐cone motifs. The N‐terminal copies within multiple‐ATP‐cones are found in a cluster (Figure 2a, spoke 1) that directly extends from the central hub. Likewise, the second copies within multiple‐ATP‐cones (Figure 2a, diamond nodes in spoke 2) extend from spoke 1, and the third copies (Figure 2a, hexagonal nodes in spoke 3) extend from spoke 2. This topology has two implications. First, it suggests that the individual copies within multiple‐ATP‐cone motifs are homologous to each other and therefore share ancestry. Furthermore, it suggests that the multiple‐ATP‐cone motifs originated from the single ATP‐cone motifs in the central hub with the N‐terminal copies retaining the greatest homology.

Previous characterization of the double ATP‐cone motif in *Pseudomonas aeruginosa* class Ia RNR had shown that only the N‐terminal copy (Figure 2a, *Pa‐1*) is functional, while the second copy (Figure 2a, *Pa‐2*) is unable to bind ligands.^25^ Based on this finding, it was proposed that the N‐terminal copy was acquired at a later time to regain allosteric regulation after the inner copy had lost function due to sequence degradation. By mapping sequence conservation onto predicted structures of multiple‐ATP‐cone motifs, we find that the ligand‐binding residues, which are highly conserved in N‐terminal ATP‐cones, are indeed more variable in the second and third copies (Figure S2). However, the previously proposed hypothesis does not account for the sequence relatedness between individual cones within multiple‐ATP‐cone motifs, which is implied by the hub‐spoke topology of our SSN. We thus propose an alternative hypothesis: that multiple‐ATP‐cone motifs were produced via a gene duplication event of an active ATP‐cone sequence. Because protein–protein interactions can be mediated by the outer N‐terminal ATP‐cone alone, we further propose that the inner copies, which were unneeded for regulation, lost their function from random mutations. This hypothesis would explain both the relatedness between inner and outer cones and the sequence variability of inner copies. Additionally, a scenario in which sequence degradation led to the loss of function of a domain, requiring subsequent recruitment (presumably via HGT) of a new functional domain is not widely observed in protein evolution and would be unlikely, assuming there was selective pressure for this enzymatic function. In contrast, domain duplication and divergence is a simpler, and well characterized, explanation.

Two other notable class I clusters are observed in the SSN. These include an island of single ATP‐cones from eukaryotic class I RNRs (Figure 2a, salmon nodes) and a cluster of single ATP‐cones from bacterial class I RNRs (Figure 2a, yellow‐green nodes) that extends from the central hub. Within the latter, the clustering of *E. coli* and *Aquifex aeolicus* ATP‐cones (Figure 2a, *Ec* and *Aa*) is interesting in light of recent studies, which report structures with two ATP molecules bound in the domain in a similar fashion. The only other ɑ subunit structure with two nucleotides bound is that of the N‐terminal ATP‐cone of *P. aeruginosa* class Ia RNR (Figure 2a, *Pa‐1*), which is in a different SSN cluster to *E. coli* and *A. aeolicus*. In the *P. aeruginosa* structure, one dATP is bound in a site that appears to be shared among all ATP‐cones, while the second dATP is in a slightly different location than the second ATP site observed in the *E. coli* and *A. aeolicus* structures. Thus, the clustering within the SSN may in part be reflecting differences in ligand binding (or the lack thereof, in the case of non‐functional ATP‐cones).

To test our evolutionary hypotheses, we performed evo‐velocity analysis on the same set of ATP‐cone sequences used in the above SSN analysis. Evo‐velocity utilizes language models to infer the fitness of individual protein sequences as well as the evolutionary direction without the construction of a phylogenetic tree.^28^ We previously applied evo‐velocity analysis on the full RNR sequences to confirm our interpretation of the RNR phylogeny.^3^ Consistent with SSN analysis, class II and III cone sequences (Figure 2c, red and yellow) cluster together and form a hub in the evo‐velocity embedded sequence space, with class I cone sequences extending from the central hub (Figure 2c, blue). When colored by “pseudotime” (a proxy measure for phylogenetic depth) (Figure 2d), evo‐velocity predicts the hub region to be the most ancestral, supporting our hypothesis that ATP‐cones in extant species have a single origin that resembles the class II and III cones. When the major SSN clusters are mapped onto the evo‐velocity network graph (Figure 2e), the two extruding heads that are more recent in pseudotime mostly correspond to the eukaryotic class I cones (Figure 2e, eukaryotes) and the inner cones from RNRs with multiple‐cone motifs (Figure 2e, spoke 2). Consistent with the hub‐spoke topology seen in SSN analysis, the inner cones are more evolutionarily distant (Figure 2d). This result further supports our hypothesis that unlike the N‐terminal cones, the inner cones were not restrained by evolutionary pressure to retain allosteric regulation and therefore were free to mutate. By a similar logic, the ancient pseudotime of class II ATP‐cones, their retention in the sequence/structure, and their relative sequence conservation, together suggest that these domains remain under selective pressure for a cryptic function, albeit one that is unlikely to be similar to the activity regulation function observed for the class I RNR cone domains.

### 2.3 The class I phylogeny provides a parsimonious model for the loss and gain of ATP‐cones

**FIGURE 3:**
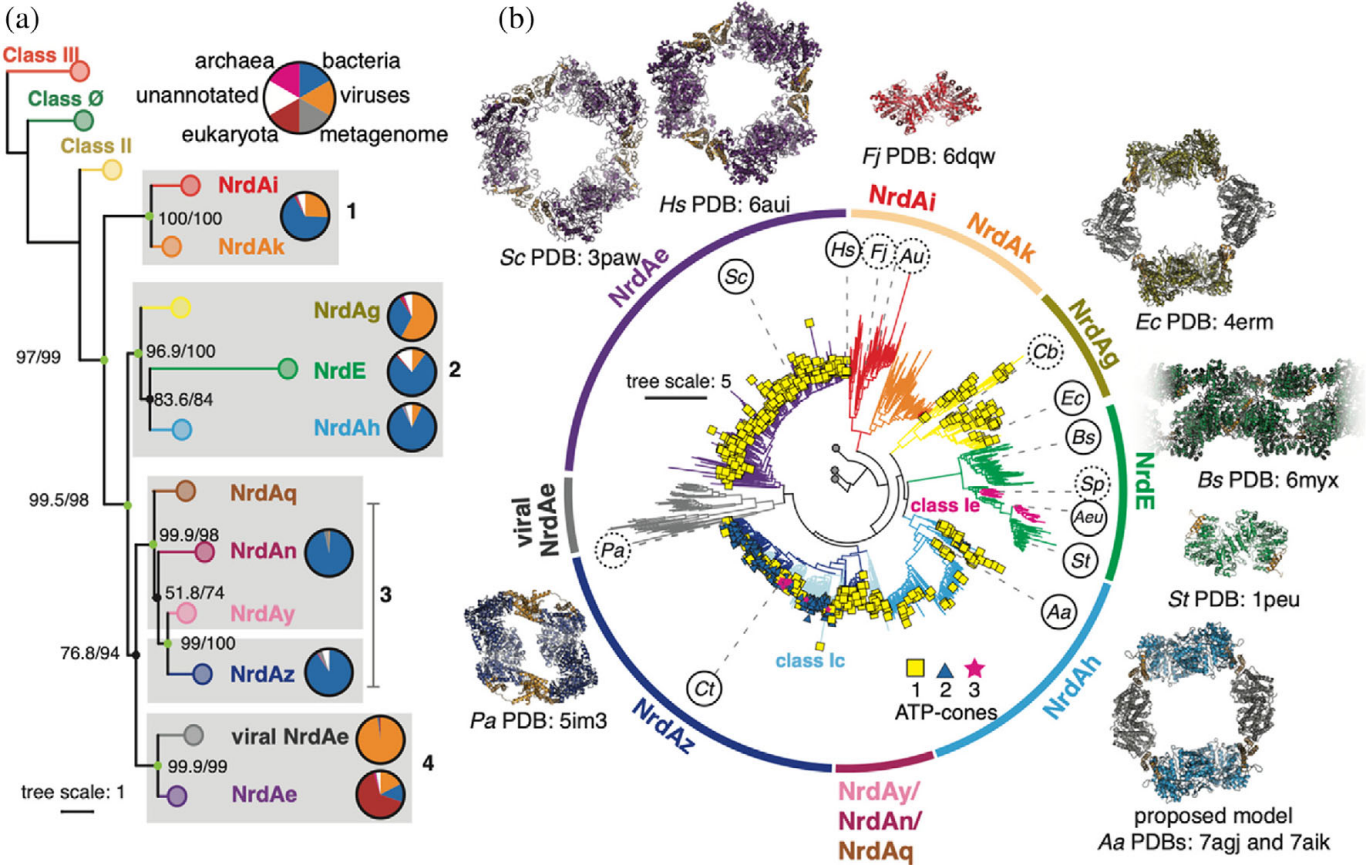
Topology of the class I ribonucleotide reductase clade provides a parsimonious model for the loss and gain of ATP‐cones. (a) Collapsed view of the class I clade pruned from the tree in Figure 1b. The tree is labeled with SH‐aLRT/UFboot2 supports on important nodes. Well supported branches (SH‐aLRT ≥ 80% and UFboot ≥ 95%) are shown as light green nodes, supports under this are black. The branches are clustered and numbered for reference in the text. The pie charts to the right of each clade show the distribution of superkingdom, where white corresponds to unannotated sequences in UniProt as of Jan 2022 release. (b) The expanded class I clade, colored as in panel (a). In Group 3 (NrdAq/An/Ay/Az), branches shown in light blue correspond to putative class Ic ɑ sequences; otherwise, they are colored in dark blue. Branches in NrdE colored in magenta are putative class Ie sequences. Tips of branches are marked by the number of ATP‐cones on the sequence, if present: one domain (yellow squares), two domains (blue triangles), and three domains (magenta stars). Class I sequences discussed in the text are indicated by circled organism IDs in clockwise order: *Hs, Homo sapiens; Fj, Flavobacterium johnsoniae; Au, Actinobacillus ureae; Cb, Clostridium botulinum; Ec, Escherichia coli*; *Bs, Bacillus subtilis; Sp, Streptococcus pyogenes; Aeu, Aerococcus urinae; St, Salmonella typhimurium; Aa, Aquifex aeolicus*; *Ct, Chlamydia trachomatis; Pa, Pseudomonas aeruginosa*; *Sc, Saccharomyces cerevisiae*. Organism IDs in dashed circles are not on the tree but their locations are approximated by sequences with ≥80% sequence identity. Classes II, III, and Ø are shown collapsed at the root. Structurally characterized class I ɑ subunits are shown in their active dimeric forms or inhibited higher oligomer forms. The ATP‐cone domain, where present, is colored in gold.

To examine possible evolutionary trajectories for the loss and gain of ATP‐cones, we next investigated the class I RNRs, which display the greatest diversity in this motif. Within class I, we resolve 11 deep‐branching clades, including a newly observed viral NrdAe clade (Figure 3a). The majority of nodes have high branch supports with the notable exception being that of NrdAn and NrdAy/NrdAz. The main consequence of this ambiguity is that NrdAq is the earliest diverging in Group 3 in half of the 20 inferences (Figure 3a), while in the other half, it is NrdAn. This is likely the result of the relatively small number of NrdAn and NrdAy sequences sampled (68 and 7, respectively, out of 2022 class I sequences in our dataset). With the exception of this ambiguity, the topology shown in Figure 3 is the most representative (8/20 inferences) (Figure S3). In Group 2, the modest branch support between NrdE and NrdAh results in some ambiguity on whether NrdAg or NrdAh is the most ancestral.

When compared to a previously reported class I α phylogeny,^4^ the most striking difference we observe is the placement of the NrdAi/Ak versus the NrdE clades (Figure S4). In the previous phylogeny, the NrdE clade diverges first from the LCA of class I, whereas in our analysis, we observe two, largely bacterial clades (NrdAi/Ak) diverging first (Figure 3a; Group 1). The NrdAi/Ak clades consist of the shortest class I α sequences: NrdAi sequences (median length of 564 aa) include biochemically characterized class Id RNRs, such as those of *Flavobacterium johnsoniae* and *Actinobacillus ureae* (Figure 3b, *Fj* and *Au*),^29^ while the NrdAk clade (median length of 620 aa) consists of yet‐uncharacterized sequences (Figure 3b). This early divergence of NrdAi/Ak sequences was supported by the majority of our topologies (17/20 inferences), whereas only one topology reconstructs NrdE as ancestral (Figure S3).

In the majority of our inferences (13/20), the NrdE clade instead emerges from a largely bacterial group of sequences that includes NrdAg and NrdAh (Figure 3a, Group 2). The NrdAg clade includes biochemically characterized class Ia sequences with a single ATP‐cone (median length of 764 aa), such as the best‐studied *E. coli* RNR (Figure 3b, *Ec*), whereas the NrdAh clade (median length of 780 aa) includes the *A. aeolicus* class Ia RNR (Figure 3b, *Aa*), which, as described above, contains an ATP‐cone that is homologous to that of the *E. coli* enzyme. Although there is some ambiguity in whether NrdAg or NrdAh diverges first from Group 2, the divergence of the NrdE clade from single ATP‐cone‐containing bacterial sequences is interesting as it is largely associated with class Ib RNRs. Class Ib α sequences are notable for having only half of the ATP‐cone motif at the N‐terminus^16^ (Figure S1a), and thus, they are shorter (median length of 718 aa) and generally lack activity regulation.^30^, ^31^ We observe two major subclades within NrdE (Figure 3b), each with one structurally characterized sequence. One subclade contains the *Bacillus subtilis* class Ib RNR (Figure 3b, *Bs*), which was shown to have evolved a convergent form of activity regulation involving its partial ATP‐cone,^17^ while the other includes the *Salmonella typhimurium* class Ib RNR (Figure 3b, *St*), which does not have activity regulation.^32^, ^33^ Interestingly, class Ie‐like sequences appear as small groups that emerge out of this second NrdE subclade (Figure 3b, magenta sequences). Class Ie RNRs utilize a dihydroxyphenylalanine (DOPA) radical in the β subunit and are currently the only known metal‐independent class I RNRs.^32^, ^33^ The placement of the class Ie RNRs within the NrdE clade demonstrates that the mechanism of radical generation can vary within a subclade.

Group 3 is comprised of largely bacterial RNRs (Figure 3a) and includes NrdAq/An/Ay/Az. Unlike the rest of the class I phylogeny, Group 3 contains many α sequences for which the corresponding β subunit contains a redox‐inert residue rather than a tyrosine as the expected radical site. Many of these class Ic‐like sequences are found in the NrdAz and NrdAq clades (Figure 3b, light blue sequences). NrdAz is also notable for having many subclades and a high occurrence of sequences with multiple ATP‐cones, such as the *P. aeruginosa* class Ia RNR and *Chlamydia trachomatis* class Ic RNR, which have 2 and 3 ATP‐cones, respectively (Figure 3b, *Pa* and *Ct*).^25^, ^34^, ^35^ Finally, in Group 4, we observe one predominantly eukaryotic clade with the majority of sequences having a single ATP‐cone (Figure 3, NrdAe) as a sibling clade to a clade of RNRs lacking ATP‐cones from eukaryote‐associated viruses (Figure 3, viral NrdAe).

Combined with our SSN and evo‐velocity analyses (Figure 2), our class I α phylogeny (Figure 3) provides a parsimonious model for the loss and gain of activity regulation. Our phylogeny supports the idea that the LCA of the class I RNRs had a single ATP‐cone. Based on this model, the short NrdAi/Ak sequences (Figure 3a, Group 1) were the first to diverge with the loss of the ATP‐cone. A single ATP‐cone was largely retained in the bacterial RNRs in Group 2. Class Ib RNRs can then be explained as having emerged from this clade (Figure 3, NrdE) as a result of gene duplication in bacteria, in which the N‐terminal ATP‐cone was truncated. In Group 3, we observe a high occurrence of multiple ATP‐cones in the NrdAz clade (Figure 3b, blue triangles or magenta stars at branch tips), which, based on our SSN analysis, we hypothesize to have initially been produced by gene duplication of a functional ATP‐cone. Finally, Group 5 is noteworthy in that it provides insight into host‐virus relationships and their possible influence on the loss and gain of ATP‐cones. While eukaryotic RNRs (NrdAe) have retained a single ATP‐cone, viral RNRs associated with eukaryotes (viral NrdAe) appear to have lost the ATP‐cone. The sequence similarity of the NrdAe and viral NrdAe RNRs is likely derived from gene exchange between viruses and hosts,^36^ while the loss of the ATP‐cone could be the result of evolutionary pressure in viruses to maintain a compact genome.^37^ Interestingly, a recent study of the Epstein–Barr virus RNR (a member of viral NrdAe) showed that although the α subunit lacks an ATP‐cone, it can regulate the activity of another enzyme.^38^

### 2.4 Long class II C‐termini contain folded domains

**FIGURE 4:**
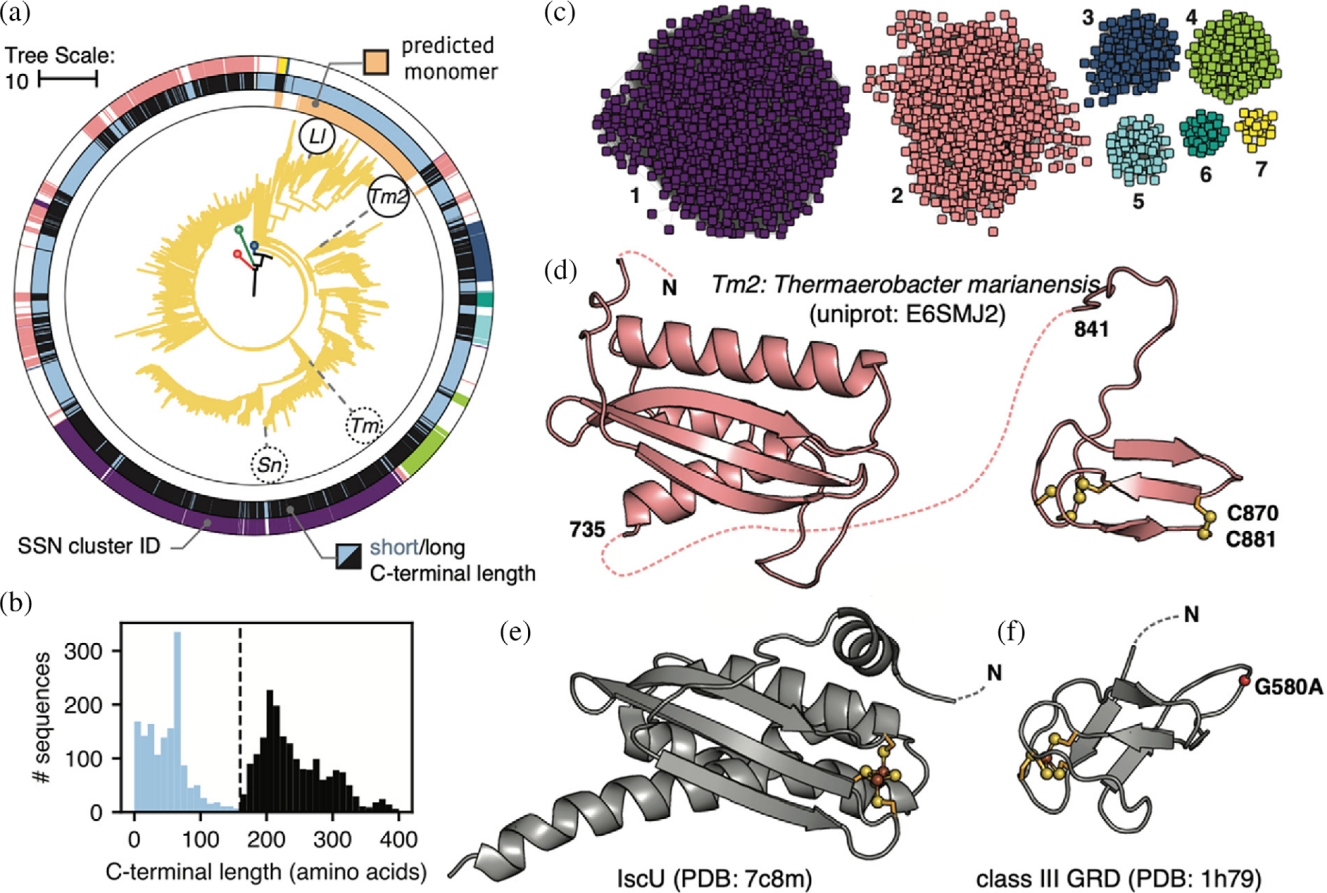
Diversity of the class II sequences is found in the C‐terminal tail. (a) Class II clade pruned from tree in Figure 1b. Sequences predicted to represent monomeric class II ribonucleotide reductases (RNRs) are shown as peach color strips in the innermost ring of the circumference. The middle color strips correspond to the length of the C‐terminal tails, where light blue is short and black is long, as depicted in panel (b). The outer color strips correspond to the SSN clusters shown in panel (c). (b) The C‐terminal length distribution is bimodal. (c) An SSN of the C‐terminal sequences. Only the largest seven clusters with the most sequences are shown for clarity. (d) Structure prediction of a representative C‐terminal sequence (*Thermoaerobacter marianensis*) from Cluster 2 (pink) in panel (c). The left domain shows structural homology to the IscU protein. The right domain is a zinc finger. (e) Crystal structure of IscU protein (PDB: 7c8m). (f) Glycyl radical domain of the class III RNR (PDB: 1h79). In panels (d–f), sulfur and iron are colored in yellow and brown, respectively. In the structure shown in panel (f), the glycine associated with radical formation was mutated to an alanine and the Cα carbon is shown as a red sphere.

We next investigated mechanisms of RNR diversification via the C‐terminus. This region, defined as the sequence after βJ (the last common fold of the catalytic barrel), varies significantly by class. Class III RNRs utilize a C‐terminal GRD for radical generation, whereas class I RNRs utilize a disordered C‐terminus with a double‐cysteine motif to reduce the oxidized active site. Although the monomeric class II RNR from *Lactobacillus leichmanii* is known to utilize a disordered C‐terminus like the class I RNRs,^18^ and certain dimeric class II RNRs are known to have long cysteine‐rich C‐terminal tails that appear to be involved in oligomerization,^23^, ^39^ very little is known about class II C‐termini in general. Interestingly, we find that class II C‐termini span a wide range of lengths with a bimodal distribution (Figure 4b). The first peak corresponds to short C‐termini (≲160 residues; a length similar to those of class I and III RNRs), while the second peak corresponds to unusually long C‐termini (160–400 residues). Based on analysis of the insertion length between βB and αB (Figure 1a, loop 1) and structure prediction by AlphaFold2, we identified the locations of monomeric and dimeric class II RNRs in our phylogeny (Figure S5 and inner color strips in Figure 4a). The majority of the monomeric class II RNRs and many of the dimeric enzymes have short C‐termini (Figure 4a, middle color strips in light blue). Sequences with long C‐termini are exclusively dimeric except for two minor subclades in the monomeric clade (Figure 4a; middle color strips in black).

To explore the classification and evolution of class II C‐termini, an SSN was constructed with the isolated C‐terminal sequences (Figure 4c). Using AlphaFold2,^40^ we predicted the structure of a representative sequence from each of the largest SSN clusters, all of which consisted of long C‐termini. Two recurring structural motifs are observed (Figure 4d). Surprisingly, the first motif is a domain with an α + β fold (Figure 4d, left) that shows structural homology with the iron–sulfur cluster assembly protein IscU when searched against the PDB (Figure 4e). The second motif at the very C‐terminus is a zinc finger domain (Figure 4d, right) as predicted previously by homology modeling.^39^ The zinc finger domain is found in the majority of dimeric class II RNRs (71%) with either a long or short C‐terminus but is only found in the two minor subclades of monomeric class II RNRs with long C‐termini.

A role for the putative IscU‐like domain has not been reported in the RNR literature. However, in the iron–sulfur cluster assembly pathway, IscU acts as a scaffold for the maturation of a [2Fe‐2S] cluster within the monomer or a [4Fe‐4S] cluster through the dimerization of two monomers.^41^ The coordination of Fe‐S clusters in IscU involves two cysteines and an aspartate, with the additional ligand being either a cysteine or histidine depending on the species. While most IscU‐like motifs in class II RNRs do not contain any cysteines at the equivalent positions, 253 out of the 600 sequences in Cluster 2 of the SSN (Figure 4a,c, salmon color strips and nodes) contain cysteines at these four positions, indicating possible shared ancestry between these residues and those from an IscU‐like protein. We hypothesize that this domain was acquired through insertion of an IscU‐like protein, which later evolved to support the functional needs of class II RNRs. The predicted structures of class II IscU‐like domains are relatively conserved, with insertion or duplication occurring in SSN Clusters 5, 6, and 7 (Figure S6). Although class II RNRs are not known to utilize Fe‐S clusters, dimerization of the IscU‐like domain may explain reports of higher order oligomerization in the dimeric class II RNRs from *T. maritima* and *Stackebrandtia nassauensis*.^23^, ^39^

All predictions of the C‐terminal zinc finger domain are structurally indistinguishable, with four cysteines poised to coordinate a Zn^2+^ ion and a cysteine pair located at the opposite end (Figure 4d, right). This domain is reminiscent of the zinc finger in the C‐terminal GRD of class III RNRs (Figure 4f). In class III RNRs, the zinc finger is thought to provide structural stability for the glycyl radical,^42^ which is located on a C‐terminal loop. Although the function of the zinc finger domain in class II RNRs remains to be explored, it may play a similar structural role by positioning the cysteine pair for active‐site reduction as suggested previously.^39^

C‐terminal sequences without a zinc finger motif were also examined. The majority of these (64%) contain two cysteines similar to class I RNRs, with smaller populations containing more than two cysteines (6%) or only one cysteine (4%). The number of residues between the double‐cysteine motif is, however, highly variable, ranging from no residue to more than four residues. In contrast, most class I RNRs show a consensus CXXC motif at the C‐terminus,^3^ with a minor population having a CXXXXC motif such as the well‐studied *E. coli* class Ia RNR. In addition, 26% of C‐terminal sequences without a zinc finger domain contain no cysteines. Roughly 40% of these represent a subclade of split class II RNRs,^43^ which consist of two chains, NrdJa and NrdJb. An AlphaFold prediction of a representative species shows that NrdJb contains the IscU‐like and zinc finger domains described above for other long C‐termini, while NrdJa contains the catalytic barrel and an extra folded domain at the very C‐terminus, which may be involved in facilitating interactions with NrdJb. Overall, it is fascinating that class II RNRs share some characteristics with class I and III RNRs in their use of zinc finger domains and/or double cysteine motifs, while the use of IscU‐like and split domains may represent strategies for oligomerization that are distinct to class II RNRs.

### 2.5 RNRs can be classified by the finger‐loop motif

Finally, we investigated an integral feature of the RNR catalytic barrel: the finger loop (defined as the insertion between βE and βF in Figure 1a). Interestingly, we find distinct sequence motifs for each major clade in our phylogeny, suggesting that they correlate with biochemical mechanism. Class Ø, I, and II RNRs all share an NXCXE motif around the catalytic cysteine (underlined) but are nevertheless distinguishable by class. In 1914 out of the 2022 class I α sequences, the finger‐loop motif is N**L** CXE, where X is primarily S/T/A. In class II, the predominant motif is N**P** CXE (3,175 out of 3,347 sequences), where X is generally G/S (with N**P** CGE found in 2005 sequences). Although the class Ø clade has not yet been biochemically characterized, it has yet another distinct finger‐loop motif of N**V** C **L**E (30 out of 35 sequences). There are no complete occurrences of this class Ø motif in either the class I or class II datasets, further indicating that this clade is phylogenetically distinct.

**FIGURE 5:**
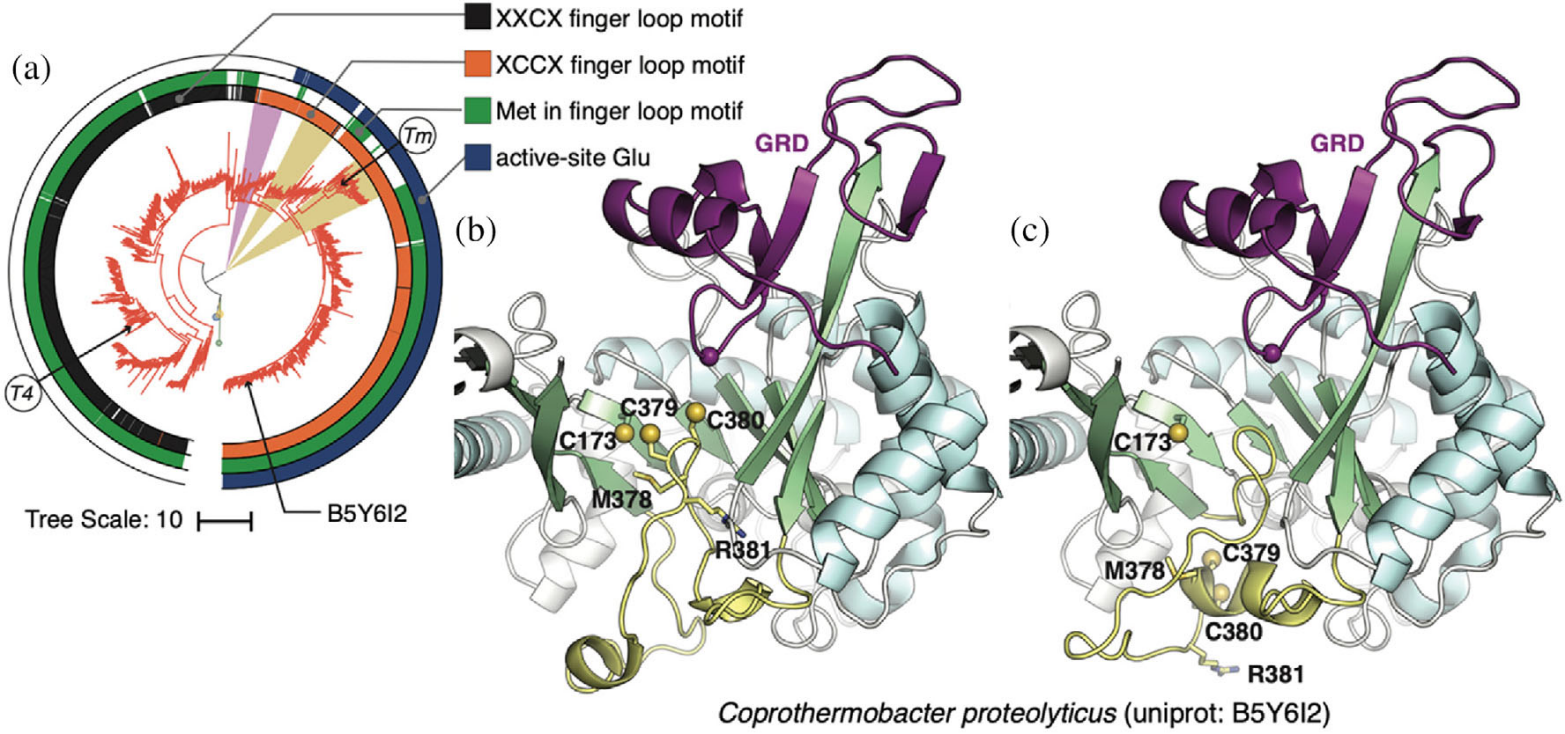
Class III diversity is driven by active‐site and finger loop motifs. (a) Class III clade pruned from tree in Figure 1b. The innermost colors trip defines the two major subclassification of class III α subunits based on the number of cystines in the finger‐loop motif: single‐cysteine (black) and double‐cysteine (orange). The middle color strips are green if the finger‐loop motif begins with a methionine. The outer color strips are blue if the active site contains a glutamate as a putative proton source. The gold wedges highlight clades where a glutamate is present but not a methionine in the finger‐loop motif, characteristics associated with only using thioredoxin as the reductant. The purple wedge highlights the subclade that neither contains a glutamate nor a methionine; these sequences have been previously subclassified as NrdD3 and thought to employ a novel reduction mechanism. Sequences with known structures are labeled as *T4, Enterobacteria* phage T4 (PDB: 1h7a) and *Tm, Thermotoga maritima* (PDB: 4u3e). (b and c) Slice‐through views of predicted models of the class III α from *Coprothermobacter proteolyticus* (UniProtID B5Y6I2 is mapped in panel [a]). The finger‐loop is colored yellow, and the cysteine sulfur atoms are shown as spheres. The glycyl radical domain is shown in purple. In panel (b), the finger‐loop is predicted to have both cysteines on the tip, whereas in panel (c), the finger loop is in the same conformation as in the *T. maritima* structure with the cysteines at the back of the barrel.

Class III RNRs, on the other hand, have a very different finger‐loop sequence. In our dataset of 1,375 class III α sequences, we find two dominant motifs: a single‐cysteine motif (MGCR) or a double‐cysteine motif (MCCR), where the methionine is highly conserved (97% and 83%, respectively). Interestingly, the divergence of these motifs occurs at a single node in our phylogeny (Figure 5a, black vs orange color strips). The two lineages are represented by the two class III RNRs, for which structures have been determined (Figure 5a, T4 and *Tm*). For both, the C‐terminal GRD is captured in the active site, such that the glycyl radical site is poised for radical transfer with the residue at the tip of the finger loop. In structures of the T4 phage class III RNR,^11^, ^44^ the finger loop contains a single cysteine at the tip (Figure S7a), which is consistent with all existing structures of class I and II RNRs and supports our current understanding that nucleotide reduction is initiated by a thiyl radical. However, in structures of the *T. maritima* class III RNR (solved independently by two research groups),^45^, ^46^ an isoleucine is instead found at the tip of the finger loop, placing the expected cysteine at the back of the catalytic barrel in an unmodeled region that contains a double cysteine motif.

To explore possible conformations of the finger loop, we employed AlphaFold2^40^ to predict structures of class III RNRs with a double‐cysteine motif. For the *T. maritima* class III sequence, AlphaFold2 predicts structures with the missing region in the finger loop modeled (Figure S7b), but otherwise identical to the crystal structure. This is expected, as the machine‐learning model enforces agreement with existing structures in the training dataset. Notably, however, the confidence score for the finger‐loop prediction is low (Figure S7c, red loop). When used to predict structures of more distant class III RNRs with double cysteines (i.e., not within the same subclade as the *T. maritima* enzyme), several or all five of the ranked models generated by AlphaFold2 (per sequence) predict the double cysteines at the tip of the finger loop (Figure 5b), while the remainder predicts the same conformation as the *T. maritima* crystal structure (Figure 5c). While it remains to be confirmed experimentally, these predictions suggest that the class III RNRs with double‐cysteine motifs can adopt the canonical finger‐loop conformation with a cysteine at the tip.

Previous biochemical studies suggest that the number of finger‐loop cysteines correlates with the mechanism of active‐site reduction in class III RNRs.^46^, ^47^ Unlike class I and II RNRs, which utilize a conserved cysteine pair on βA and βF to provide two electrons for nucleotide reduction, only the cysteine in βA is highly conserved (92.6%) in class III RNRs. For *E. coli* class III RNR, it was shown that the methionine in its single‐cysteine MGCR motif plays an important role in formate‐dependent nucleotide reduction by forming a thiosulfuranyl radical with the conserved cysteine on βA.^47^ This would explain the fact that the methionine is conserved (97%) in this subclass of enzymes. In the case of *Neisseria bacilliformis* class III RNR, it was shown that one of the cysteines in its SCCR motif (lacking the upstream methionine) forms a disulfide bond with the βA cysteine in a manner similar to class I and II RNRs to donate two electrons, which is subsequently reduced by thioredoxin.^46^ However, from our analysis, the majority (83%) of class III sequences with double‐cysteine motifs contain a methionine at the start of the motif and thus appear to contain essential components for utilizing either formate or thioredoxin as reducing equivalents. When mapped onto our tree, the methionine‐containing sequences appear to be ancestral, consistent with the usage of formate as an electron donor in anaerobic metabolism (Figure 5a; green color strips). As noted previously,^48^ the use of formate links the class III RNRs with the glycyl radical enzyme (GRE) pyruvate‐formate lyase and suggests a common origin for the structurally related RNR and GRE families.

In the study of *N. bacilliformis* class III RNR,^46^ it was further proposed that an active‐site glutamate that is at an equivalent position to the established proton source in *E. coli* class Ia RNR (in the reduction of the 3′‐keto‐deoxynucleotide intermediate) is essential for formate‐independent nucleotide reduction. By comparing where sequences with this glutamate and the finger‐loop methionine map on our tree, we identified two small subclades, one of which includes the enzymes from *N. bacilliformis* and *T. maritima*, that contain glutamate but do not have methionine and are thus likely to solely utilize thioredoxin as the reductant (Figure 5a, orange wedges). We also observed the clade previously classified as NrdD3 that contains a QCCR/A motif with neither the methionine nor glutamate (Figure 5a, purple wedge), which has been suggested to employ a novel reduction mechanism.^46^ Overall, our analysis supports the idea that the finger‐loop sequence is closely coupled to biochemical mechanism.

## 3 DISCUSSION

Previously, we reported the first large‐scale phylogenetic inference of the RNR family using the conserved 10‐stranded α/β barrel to align the highly diverged sequences of the catalytic α subunit. In this study, we sought to understand the mechanisms of diversification by examining extensions and insertions about the barrel. We first examined the N‐terminal ATP‐cone extensions, which are involved in allosteric regulation but are not essential for RNR activity. Despite being non‐essential, we found surprising patterns of sequence homology that suggest that the ATP‐cone had a single origin and that it was inherited vertically, rather than by HGT. Among the family, the class I ATP‐cones have diverged the most, showing the greatest diversity, possibly because these enzymes require a second subunit for turnover and are able to make use of ATP‐cones for oligomerization‐dependent activity regulation. We next examined the C‐terminal extensions, which are important for RNR activity but are not directly involved in the key step of nucleotide reduction. We find that the C‐terminal extensions vary by class, suggesting that they evolved to support class‐specific catalytic mechanisms. Class I RNRs almost exclusively contain a C‐terminal double‐cysteine motif for active‐site re‐reduction, while class III RNRs contain a C‐terminal GRD for the generation of the initial thiyl radical. Class II RNRs, on the other hand, share certain traits with class I and III RNRs (such as the use of cysteines and a zinc finger fold), while others are totally unique to the class (such as the use of an IscU‐like domain). The variability within class II is interesting and suggests that the tail may have multiple roles in addition to re‐reduction, such as oligomerization. Finally, we examined the finger‐loop insertion, which is essential for the generation of the thiyl radical. Although expected, we were still surprised to find that the finger‐loop sequence is highly conserved within each class, indicating that it is tightly coupled to the catalytic mechanism. Here, class III RNRs showed the greatest diversity with two distinct motifs that enable this class to utilize different external reductants. Overall, our work demonstrates that mechanisms of diversification within an enzyme family can be evaluated by examining patterns of homology within extensions and insertions both at the class level and at the enzyme family level.

While N‐terminal ATP‐cone motifs appear to have been gained via vertical inheritance, our analyses suggest that there are several mechanisms for the loss of ATP‐cones. The lack of ATP‐cones observed in the majority of class II RNRs suggests that there was a lack of evolutionary pressure to retain this function, possibly because this class evolved to use a cofactor for catalysis rather than a separate protein. Similarly, the inner copies of multiple‐ATP‐cone motifs appeared to have drifted from the functionally active form, likely because they do not participate in oligomerization. Interestingly, the viral NrdAe RNRs lack ATP‐cones despite sharing homology with their host RNRs (NrdAe), which have ATP‐cones. Likewise, the minimal class Ø RNRs, which are associated with cyanophages, also lack ATP‐cones. Thus, another mechanism for the loss of an ATP‐cone may be evolutionary pressure for viruses to maintain compact genomes. At the same time, evidence for evolutionary pressure to compel activity regulation can also be seen in RNRs lacking ATP‐cones. One example is the *B. subtilis* class Ib RNR, where new ligand‐binding sites appeared to have evolved within the truncated ATP‐cone.^17^ Additionally, some class Id and Ib RNRs have been reported to contain ATP‐cones on the β subunit.^49^ In our dataset, we find that ATP‐cones exist on the β subunit almost exclusively where the operon is organized such that α is transcribed downstream from β (Figure S8), contrasting with the majority of the class I RNR operons, which are organized with α upstream from β. We thus hypothesize that the β subunit may have acquired an N‐terminal ATP‐cone through gene insertion in the middle of an ATP‐cone‐containing α sequence. We observe an especially high occurrence of operons organized as β‐α in the NrdAi (class Id) clade (70/104 sequences).

**FIGURE 6:**
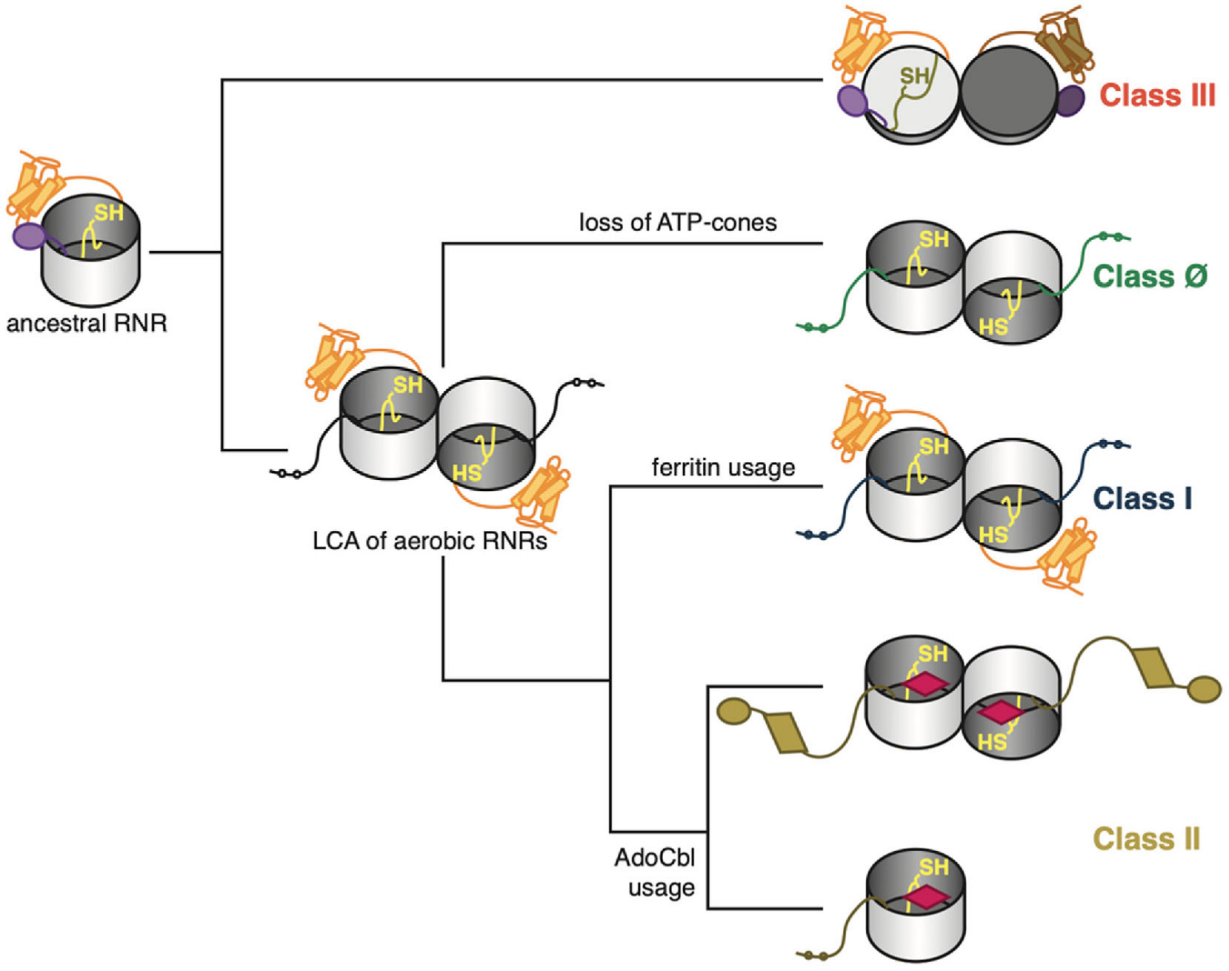
Proposed schematic of ribonucleotide reductase (RNR) functional evolution. The ancestral RNR likely resembled a glycyl radical enzyme with an N‐terminal ATP‐cone (orange bundle), a finger loop (yellow), and a C‐terminal glycyl radical domain (purple ellipse). In class III RNRs, the barrels dimerize such that the active sites point in opposite directions. The last common ancestor of the aerobic RNRs likely had an N‐terminal ATP‐cone and a cysteine‐rich C‐terminus, possibly recruiting a ferritin subunit for radical generation. The aerobic RNRs further specialized into ferritin or AdoCbl (magenta diamond) usage. ATP‐cones were lost in the class Ø RNRs and largely lost in the class II RNRs.

Based on our previous work,^3^ and our work here, we propose an evolutionary model in which the LCA of the modern RNR had a single N‐terminal ATP‐cone, which then diversified into distinct biochemical classes via the C‐terminal extensions and insertions (Figure 6). The oxygen‐sensitive class III RNRs diverged first, making use of a C‐terminal GRD and multiple strategies for active‐site reduction encoded in the finger‐loop sequence. The increasing presence of oxygen led to the emergence of the LCA of the oxygen‐tolerant and oxygen‐dependent classes (Ø, I, II), from which the class Ø RNRs diverged with the loss of an ATP‐cone. Consistent with the idea that they descend from a common ancestor, the class Ø, I, and II share similar yet class‐specific finger‐loop sequences and cysteine‐rich C‐terminal extensions. While class I RNRs appear to use a common mechanism for active‐site reduction, they display diversity in mechanisms of activity regulation and radical generation, as seen by the various types of N‐terminal ATP‐cones and cofactors in the ferritin subunit. In contrast, class II RNRs use a common cofactor for catalysis but are diversified by the C‐terminal extension, in ways that we do not yet fully understand. Despite the incredible complexity of the RNR family, we have shown that by analyzing extensions and insertions with multiple types of bioinformatic analyses and structure prediction, we can propose an evolutionary model and also uncover new motifs and patterns for future investigation.

## 4 METHODS

### 4.1 Analysis of ATP‐cones

The presence of ATP‐cone motifs in our dataset of 6,779 RNR α sequences was determined by alignment against Pfam 03477 with HMMER using an E‐value cutoff of 10^−3^. The domains were then mapped onto the RNR ɑ subunit phylogeny to investigate the ubiquity and evolution of the ATP‐cone across phyla.^50^, ^51^ For the SSN of the ATP cones, ATP cone sequences identified by InterPro^52^ in the tree was extracted and used to construct an SSN using EFI‐EST.^53^ The resulting SSN was filtered with an alignment score cutoff of 20.5, which corresponds to an approximate E‐value of 10^−20^.

We note that a number of long class I sequences with multiple ATP‐cones were misannotated as class II sequences in the UniProtKB database.^54^ Upon closer examination of the *nrd* operons, these misannotated *nrdA* genes are associated with a gene for a class I radical‐generating subunit (*nrdB*) immediately downstream. In our phylogeny, these sequences appear in the class I clade.

### 4.2 Analysis of class II C‐termini

To analyze the C‐terminal extensions, the sequences from the class II clade in the tree were aligned with MAFFT.^55^ Due to the absence of the sequence of the only structurally characterized dimeric class II RNR from *T. maritima* in the tree, a pair‐wise alignment between the sequence of *T. maritima* class II RNR (Uniprot: O33839) and its closest homolog in the dataset (93% identical), *Thermotoga neapolitana* class II RNR sequence (Uniprot: B9K714), was performed with Clustal Omega.^56^ Alignment columns in the class II MSA after the column that corresponds to residue 639 in B9K714 (630 in O33839) were then assigned as the C‐termini of the corresponding sequences. The extracted C‐termini sequences were used to construct an SSN using EFI‐EST,^53^ and an alignment score of 30 was used to filter the edges, corresponding to an approximate E‐value of 10^−30^. The DALI server^57^ was used to identify the homology of folded domains in AlphaFold2 predictions of the C‐termini.

### 4.3 Analysis of class III finger loop motifs

The sequences from the class III clade in the tree were aligned with MAFFT.^55^ Alignment columns corresponding to residues 319–367 in *T. maritima* class III RNR (Uniprot: Q9WYL6) were assigned as the finger‐loop region and extracted. Insertions within the finger loop in a small number of extracted sequences (22 out of 1,375) were manually removed, and the sequences were then realigned with MAFFT. Columns corresponding to the finger‐loop motif were determined by alignment with residues 328–331 (SCCR) in Q9WYL6. The column corresponding to the proton donor glutamate was determined by alignment with residue 495 in Q9WYL6.

### 4.4 AlphaFold predictions

For all AlphaFold predictions, sequences of interest were retrieved from UniProt^54^ and input for AlphaFold2 prediction^40^ (version 2.0.0) with the five default model parameters and a template date cutoff of May 14, 2020.

## AUTHOR CONTRIBUTIONS

**Audrey A. Burnim:** Data curation (lead); formal analysis (lead); investigation (equal); visualization (lead); writing – original draft (equal); writing – review and editing (equal). **Da Xu:** Data curation (lead); formal analysis (lead); investigation (lead); visualization (equal); writing – original draft (equal); writing – review and editing (equal). **Matthew A. Spence:** Formal analysis (equal); writing – review and editing (equal). **Colin J. Jackson:** Formal analysis (equal); methodology (equal); project administration (equal); supervision (equal); writing – review and editing (equal). **Nozomi Ando:** Conceptualization (lead); formal analysis (equal); funding acquisition (equal); investigation (lead); project administration (lead); supervision (lead); writing – original draft (equal); writing – review and editing (equal).

## Supporting information


**Appendix S1:** Supporting Information.

## Data Availability

The data that support the findings of this study are available from the corresponding author upon reasonable request.
