# Supplementary material for: Analysis of insertions and extensions in the functional evolution of the ribonucleotide reductase family
Source: Protein Sci. 2022 Dec;31(12):e4483. doi: 10.1002/pro.4483 (PMC9669993; doi:10.1002/pro.4483)
Supplement: Supplementary file 1 — Appendix S1: Supporting Information. [file PRO-31-e4483-s001.pdf]

Analysis of insertions and extensions in the functional evolution of the ribonucleotide reductase family

Audrey A. Burnim[1]*, Da Xu[1]*, Matthew A. Spence[2], Colin Jackson[2,3,4], and Nozomi Ando[1†]

[1]Department of Chemistry and Chemical Biology, Cornell University, Ithaca, NY 14850, USA. [2]Research School of Chemistry, Australian National University, Canberra, ACT, 2601, Australia. [3]Australian Research Council Centre of Excellence for Innovations in Peptide and Protein Science, Australian National University, Canberra, ACT 2601, Australia. [4]Australian Research Council Centre of Excellence in Synthetic Biology, Australian National University, Canberra, ACT 2601, Australia.

* These authors contributed equally to this work.

[†] Correspondence should be addressed to nozomi.ando@cornell.edu.

Figures S1-S8

Supporting References

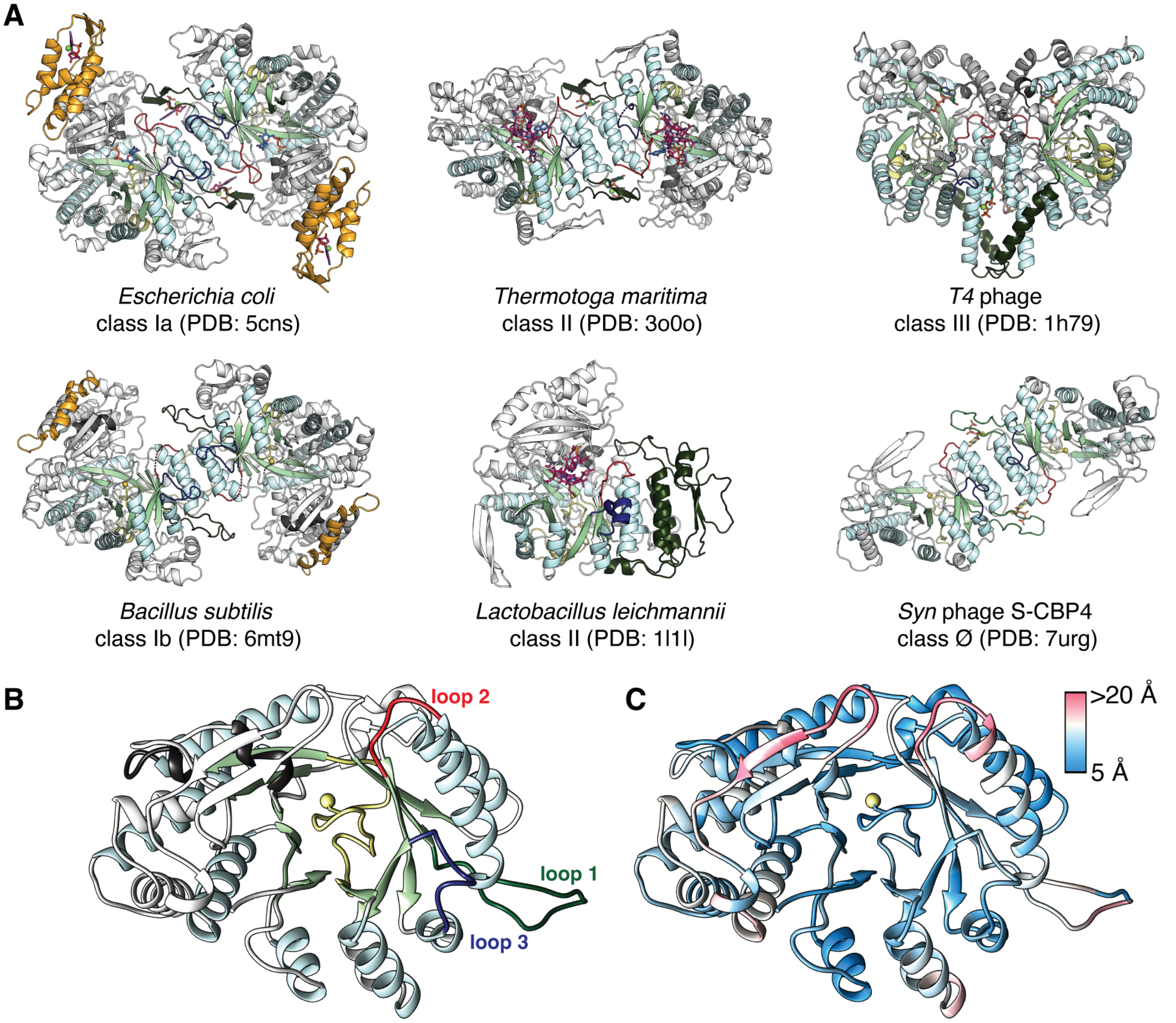

Figure S1. All RNRs share a structurally homologous catalytic barrel. (**A**) Representative structures of the class I α subunit include those of the *E. coli* class Ia RNR, which has a single N-terminal ATP-cone domain (orange) and the *B. subtilis* class Ib RNR, which as a partial N-terminal ATP-cone (orange). Class II RNRs can be dimeric like the *T. maritima* enzyme or monomeric like the *L. leichmannii* enzyme, which has a long loop 1 insertion (dark green) that mimics a dimer interface. The AdoCbl cofactor is shown as magenta sticks. Class III RNRs, such as that from T4 phage, dimerize in a different orientation than class I and II RNRs, facilitated by a long helix dark green) in place of loop 1. The class Ø RNRs are the most minimal RNRs structurally characterized to-date. The class Ø dimer from *Synechococcus* phage S-CBP4 is shown. (**B**). Shown here is one monomer of the *Synechococcus* phage S-CBP4 α subunit (PDB: 7urg), colored as in panel A and Figure 1A. (**C**) The structure in panel B is shown colored according to the RMSD of all 12 non-redundant RNR structures reported to-date. Despite significant diversity in the RNR family, the core fold is conserved, as indicated by the low RMSD values for secondary structure elements in the catalytic barrel.

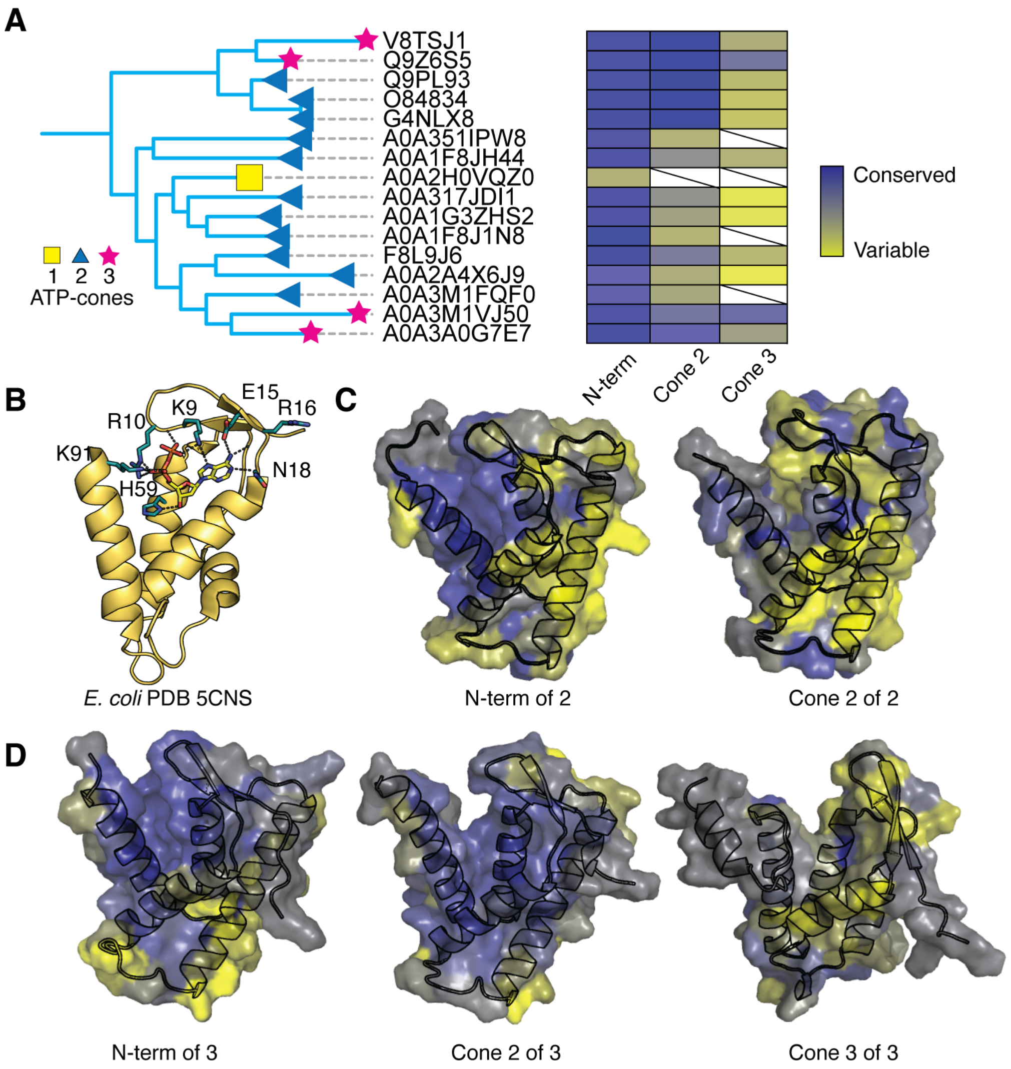

Figure S2. Inner copies in tandem ATP-cones diverge from the canonical motif while N-terminal cones maintain homology. (**A**) A tandem ATP-cone containing subset of the NrdAz clade pruned from Figure 1B. Tips denote number of ATP-cones in the sequence. The color strips show heatmaps of the individual bit score of each copy of the tandem ATP-cones when aligned to the Pfam03477 HMM. (**B**) For comparison, the ATP-cone from *E. coli* class Ia α is shown with dATP bound (yellow). Key interacting residues are shown as teal sticks. (**C, D**) AlphaFold2 models of ATP-cone domains from the (C) double- and (D) triple-domain containing sequences, UniProt IDs A0A2A4X6J9 and A0A1F8J1N8 (C, *Chlamydiae* bacterium) and (D, *Candidatus Aerophobetes* bacterium). Sequence conservation was calculated separately for all sequences in our dataset with two or three ATP-cones in Consurf.[1] Conservation scores were then mapped onto the AlphaFold2 models, where residues of high conservation are purple and low conservation are yellow.

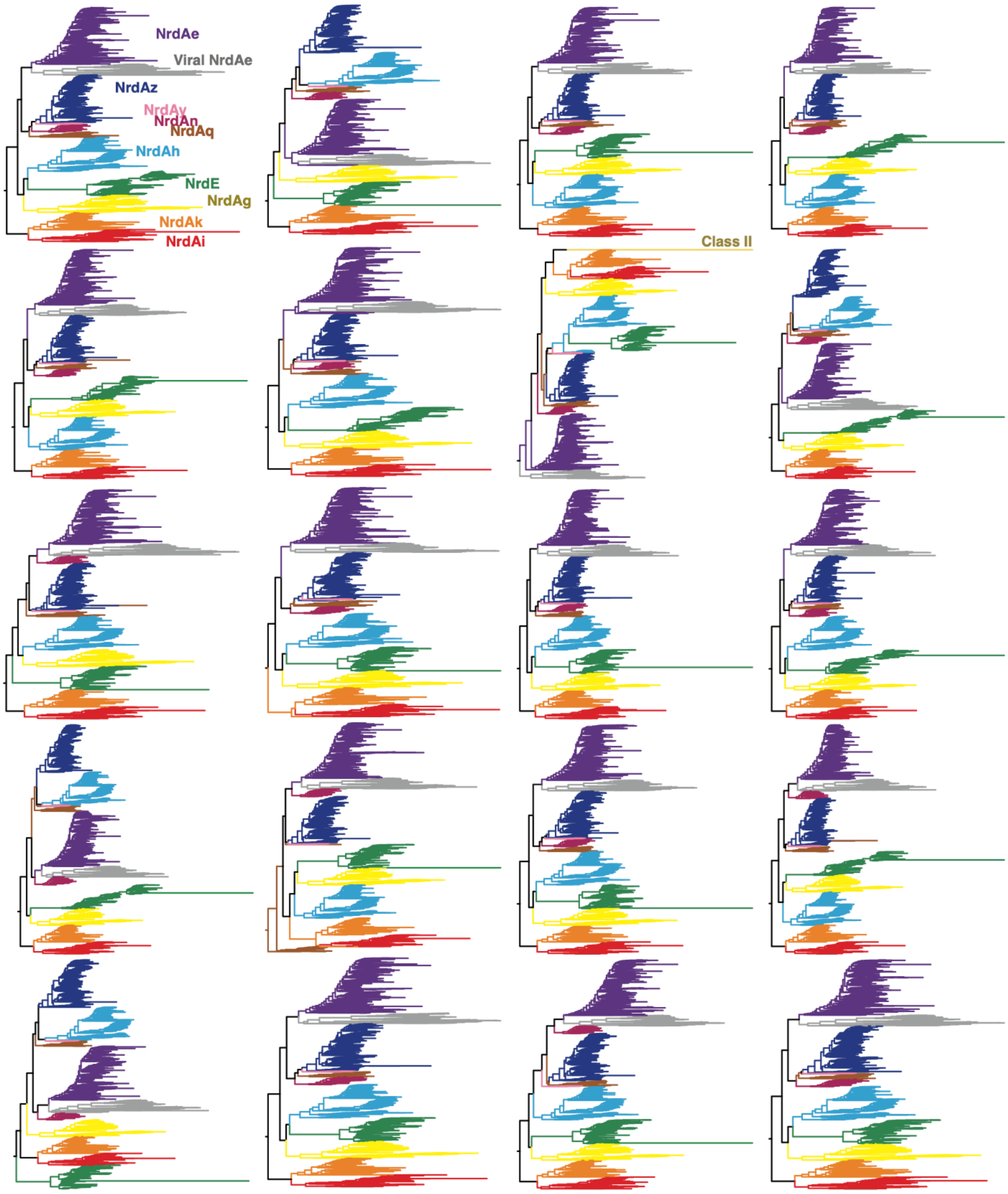

Figure S3. All 20 topologies of the class I clade. The first ten trees from left to right, top to bottom, were calculated with the LG+R10 model of evolution, and the latter ten trees were calculated with the WAG+R10 model of evolution.

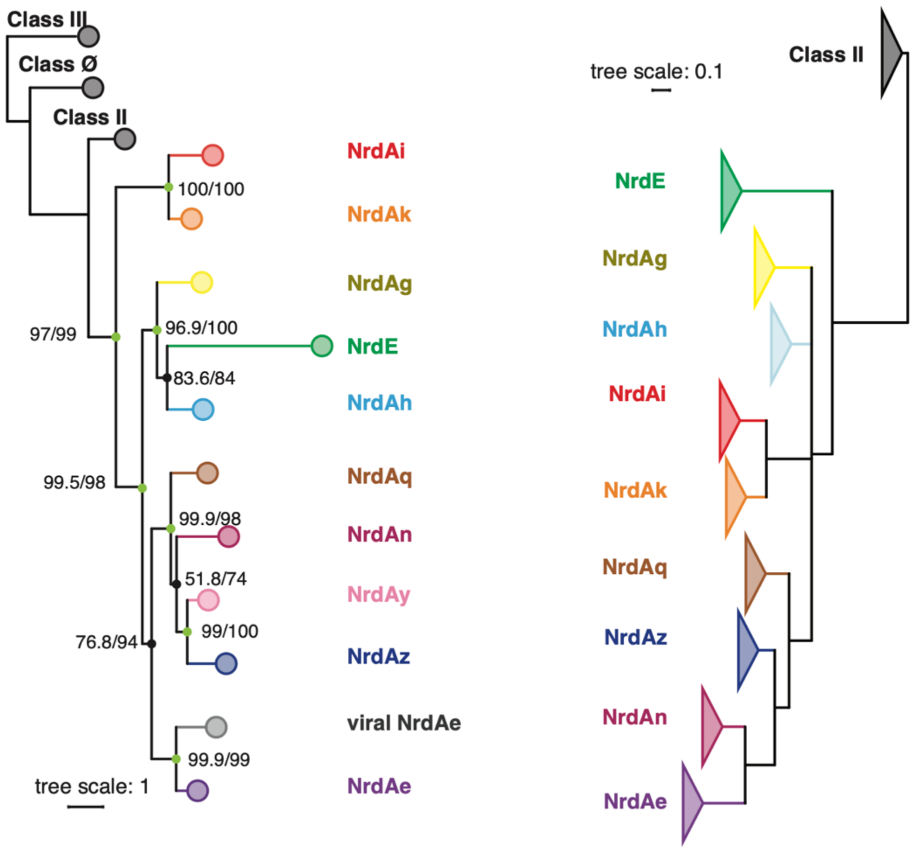

Figure S4. Comparison of class I RNR phylogenies. Left: the same as shown in Figure 3A. Right: the tree published by Martínez-Carranza, *et al.*[2] shown in collapsed form for direct comparison.

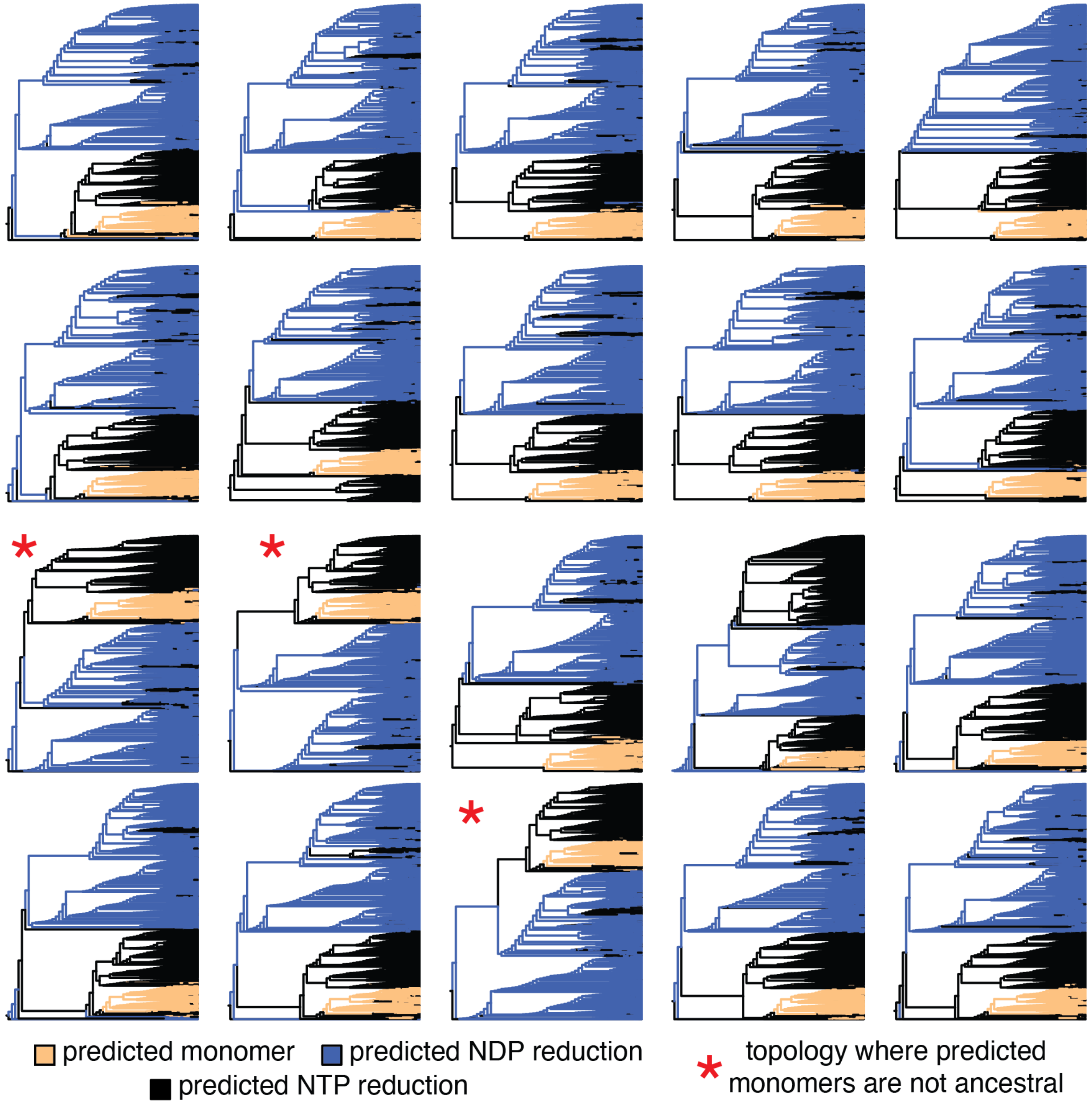

Figure S5. A subclass of class II ribonucleotide reductases mimic dimerization with an extended insertion at loop 1 (see Figure S1). The topologies of the class II clade from all 20 tree inferences. The top ten trees were calculated un-der the LG+R10 model of evolution, and the bottom ten trees were calculated under the WAG+R10 model of evolution. The peach branches correspond to sequences that contain the monomer-conferring 29-82 residue insertion between βB and αB in an MSA of class II RNRs and are in clades that have a representative sequence predicted by AlphaFold2 to be monomeric. Unlike class I and III RNRs which are specific for ribonucleoside diphosphates (NDPs) and triphosphates (NTPs), respectively, class II RNRs show diversity in substrate preference. Substrate preference is associated with a 4-5 residue sequence motif preceding the catalytic barrel (Figure 1A, left star), with PNSP (NDP-specific) and PAGR (NTP-specific) being the two most predominant patterns.[3,4] Blue branches are sequences that are predicted to be specific for NDP reduction and black branches are sequences that are predicted to reduce NTPs. Note that all predicted monomer sequences are predicted to be NTP-specific. The three outlier topologies that do not predict the ancestral branch to the class II clade as NTP-specific monomer enzymes are annotated with a red asterisk.

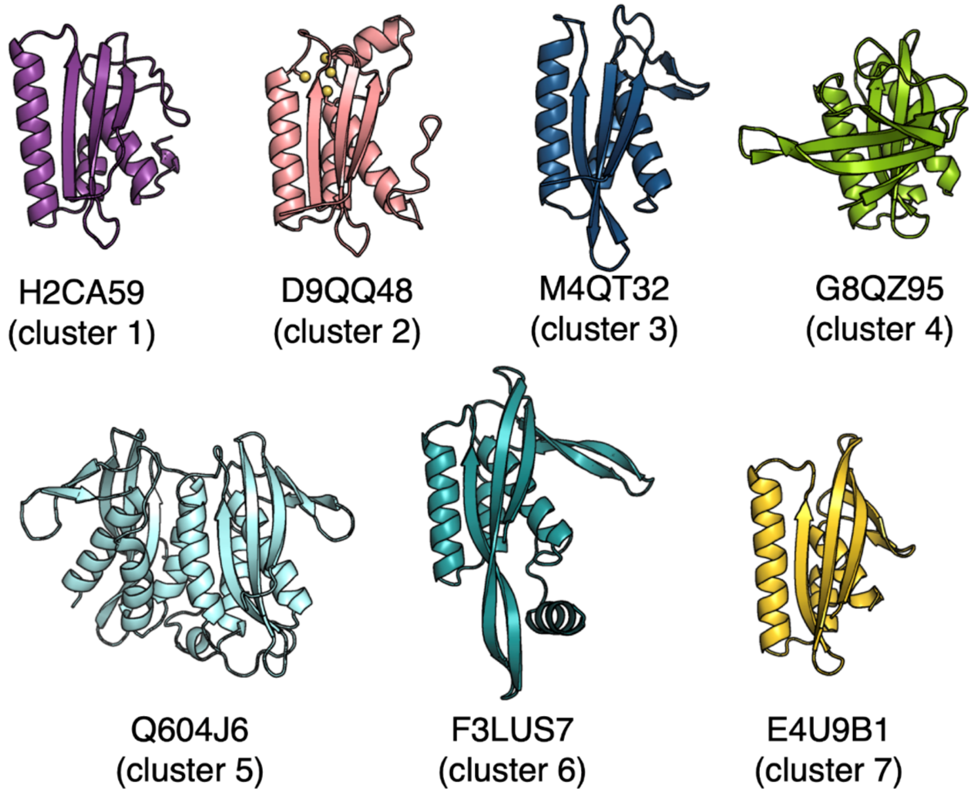

Figure S6. AlphaFold2 predictions of IscU-like C-terminal domains from representative sequences in each major cluster of the SSN shown in Figure 4C. The UniProt IDs are shown for each sequence. In order of cluster number, the sequences correspond to the organisms *Leptonema illini* (H2CA59), *Acetohalobium arabaticum* (D9QQ48), *Loktanella* phage pCB2051-A (M4QT32), *Owenweeksia hongkongensis* (G8QZ95), *Methylococcus capsulatus* (Q604J6), *Rubrivivax benzoatilyticus* JA2 (F3LUS7), and *Oceanithermus profundus* (E4U9B1), respectively.

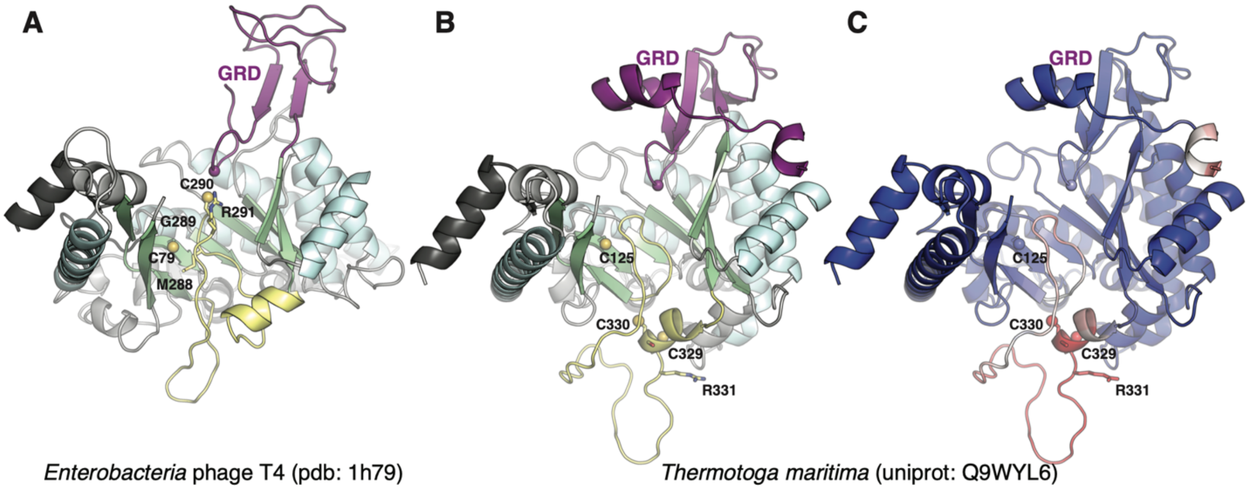

Figure S7. Class III sequences with crystal structures. The finger-loop is shown in yellow, and the cysteine sulfur atoms are shown as spheres. The glycyl radical domain (GRD) is shown in purple, and the alpha-carbon of the radical site is shown as a sphere. (**A**) Structure of class III RNR from *Enterobacteria* phage T4 (PDB: 1h79). A single cysteine is at the tip of the finger loop. (**B**) AlphaFold2 prediction of *Thermotoga maritima* is identical to the known structure (PDB: 4u3e) except with the missing region in the finger loop modeled. (**C**) Model in panel B colored by AlphaFold pLDDT confidence score where red is low confidence (pLDDT = 13.72) and blue is high (pLDDT = 100.96).

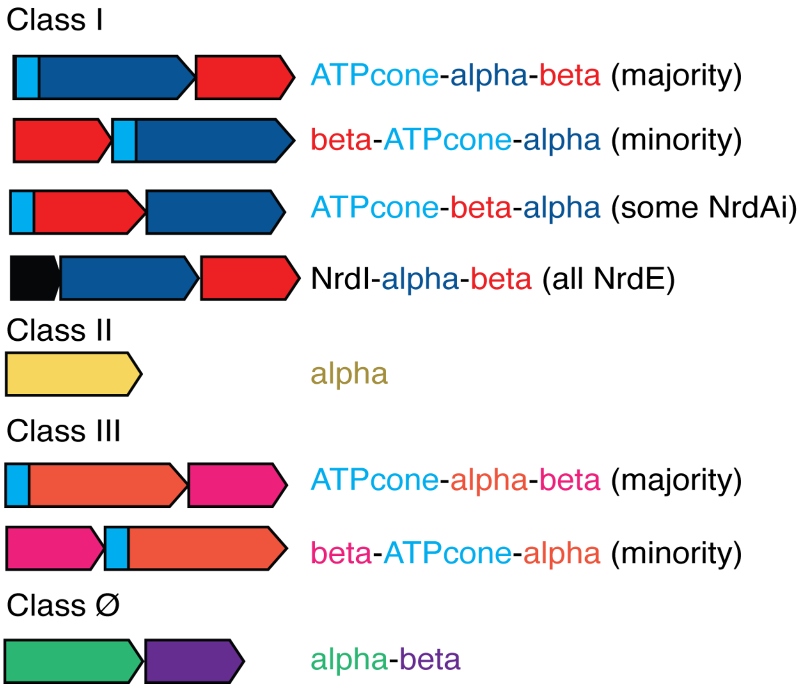

Figure S8. Overview of RNR operon organization. For sequences that contain ATP-cones, the location is shown in light blue.

Supporting References

1. Landau M, Mayrose I, Rosenberg Y, Glaser F, Martz E, Pupko T, Ben-Tal N (2005) ConSurf 2005: the projection of evolutionary conservation scores of residues on protein structures. Nucleic Acids Res. 33: W299-W302.

2. Martínez-Carranza M, Jonna VR, Lundin D, Sahlin M, Carlson L-A, Jemal N, Högbom M, Sjöberg B-M, Stenmark P, Hofer A (2020) A ribonucleotide reductase from Clostridium botulinum reveals distinct evolutionary pathways to regulation via the overall activity site. J. Biol. Chem. 295:15576–15587.

3. Schell E, Nouairia G, Steiner E, Weber N, Lundin D, Loderer C (2021) Structural determinants and distribution of phosphate specificity in ribonucleotide reductases. J. Biol. Chem.:101008.

4. Wei Y (2015) Mechanistic Investigations of Class III Anaerobic Ribonucleotide Reductases. [Doctoral Dissertation, Massachusetts Institute of Technology, Cambridge, MA]
